# Comparison of different strategies for using fossil calibrations to generate the time prior in Bayesian molecular clock dating

**DOI:** 10.1016/j.ympev.2017.07.005

**Published:** 2017-09

**Authors:** Jose Barba-Montoya, Mario dos Reis, Ziheng Yang

**Affiliations:** a Department of Genetics, Evolution and Environment, University College London, Gower Street, London WC1E 6BT, UK; b School of Biological and Chemical Sciences, Queen Mary University of London, Mile End Road, London E1 4NS, UK

**Keywords:** Bayesian inference, Molecular clock dating, Divergence times, Fossil calibration, Time prior

## Abstract

Fossil calibrations are the utmost source of information for resolving the distances between molecular sequences into estimates of absolute times and absolute rates in molecular clock dating analysis. The quality of calibrations is thus expected to have a major impact on divergence time estimates even if a huge amount of molecular data is available. In Bayesian molecular clock dating, fossil calibration information is incorporated in the analysis through the prior on divergence times (the time prior). Here, we evaluate three strategies for converting fossil calibrations (in the form of minimum- and maximum-age bounds) into the prior on times, which differ according to whether they borrow information from the maximum age of ancestral nodes and minimum age of descendent nodes to form constraints for any given node on the phylogeny. We study a simple example that is analytically tractable, and analyze two real datasets (one of 10 primate species and another of 48 seed plant species) using three Bayesian dating programs: MCMCTree, MrBayes and BEAST2. We examine how different calibration strategies, the birth-death process, and automatic truncation (to enforce the constraint that ancestral nodes are older than descendent nodes) interact to determine the time prior. In general, truncation has a great impact on calibrations so that the effective priors on the calibration node ages after the truncation can be very different from the user-specified calibration densities. The different strategies for generating the effective prior also had considerable impact, leading to very different marginal effective priors. Arbitrary parameters used to implement minimum-bound calibrations were found to have a strong impact upon the prior and posterior of the divergence times. Our results highlight the importance of inspecting the joint time prior used by the dating program before any Bayesian dating analysis.

## Highlights

• Fossil calibrations are the utmost source of information in molecular clock dating.
• The quality of calibrations has a major impact on divergence time estimates.
• In general, truncation has a great impact on calibrations.
• The different strategies for generating the effective prior also had considerable impact.
• It is important to inspect the joint time prior used by the dating program before any Bayesian dating analysis.

## Graphical abstract

**Figure:**
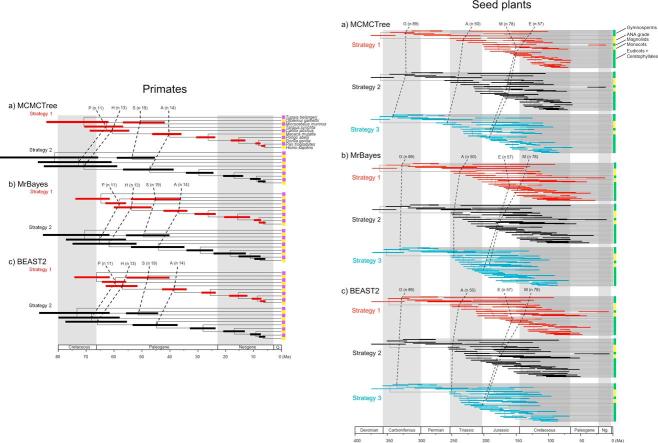

## 1 Introduction

Bayesian inference has become the methodology of choice for molecular clock dating of species divergences because it provides a natural framework for incorporating different sources of information (e.g., from fossils and molecules) (dos Reis et al., 2016). In a Bayesian dating analysis, one would ideally summarize the relevant prior evidence about species divergence times (say, from the fossil record, geological events, etc.) in a multidimensional joint prior of ages for all nodes on the phylogeny (called the time prior). However, specifying high-dimensional priors with complex correlation structures is a notoriously difficult task, and furthermore, our knowledge of the fossil evidence and of how it informs the species divergence times is very imprecise. The current practice is for the paleontologist to specify minimum- and maximum-age constraints on certain nodes on the tree based on the fossil evidence (Thorne et al., 1998, Kishino et al., 2001, Benton et al., 2009, Ho and Phillips, 2009). Such *user-specified* fossil calibrations are then used by the Bayesian dating program to construct the time prior, with the distribution of the ages of non-calibration nodes supplanted by a branching-process model (e.g., a birth-death process) (Yang and Rannala, 2006). The user-specified calibration densities are assigned to single nodes on the tree and often do not satisfy the requirement that any ancestral node should be older than its descendants, and thus the dating software must ‘truncate’ the calibration densities to satisfy this constraint. We refer to the resulting prior of node ages used by the dating software as the *effective prior*, and this may be very different from the original user-specified calibration densities (Inoue et al., 2010, Warnock et al., 2015). Furthermore, Bayesian dating programs such as MultiDivTime (Thorne et al., 1998), MCMCTree (Yang, 2007), BEAST2 (Bouckaert et al., 2014) and MrBayes (Ronquist et al., 2012b) use different procedures to combine calibration densities with the birth-death process model to generate the time prior, so that different programs may produce very different time priors from the same user-specified fossil calibrations (Inoue et al., 2010).

Thus, users of dating software are encouraged to run the Markov Chain Monte Carlo (MCMC) algorithm without molecular data to generate the time prior used by the program and to inspect it to ensure that it is a reasonable representation of the fossil evidence. A cross-validation method for assessing the quality of calibrations, based on the consistency between fossils and between fossils and molecules, has also been proposed (Near et al., 2005). This was noted to sometimes lead to the selection of calibrations of poor reliability (Marshall, 2008, Benton et al., 2009, Warnock et al., 2015). The problem appears to be partly due to the fact that fossil-calibration constraints provided by the paleontologist are “over-interpreted” by the Bayesian dating program. For example, when fossil evidence suggests that the age of a clade is between 50 Ma and 100 Ma, the dating software may incorporate that information by assigning a uniform distribution, *t* ∼ U(50, 100), implying, for example, P{50 < *t* < 60} = P{90 < *t* < 100}. Such probabilistic statements about the true age may not be intended by the paleontologist. However minimum and maximum bounds alone, in the form of 50 < *t* < 100, are insufficient to permit a Bayesian dating analysis: a full statistical distribution for the true age has to be specified.

**Fig. 1:**
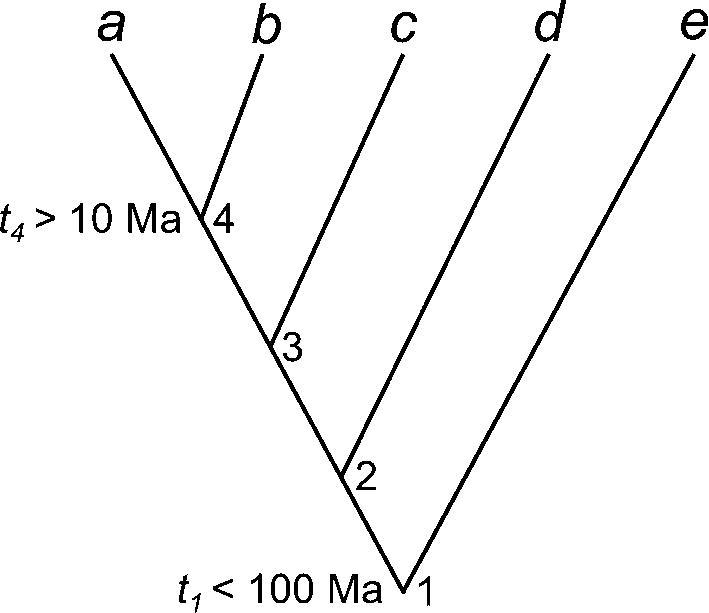
A five-species phylogeny used in the analytical example of fossil calibration strategies.

The way that the fossil-based bounds on node ages are converted into statistical distributions in a dating analysis may thus have an important impact on the posterior time estimates. Consider the unbalanced 5-species phylogeny of Fig. 1. Suppose that fossil evidence suggests that the age of node 4 should be at least 10 Myrs, while the age of the root is at most 100 Ma, with *t*_4_ > 10 and *t*_1_ < 100 (Fig. 1). Three simple strategies appear possible to construct the calibration densities. In strategy 1 (st1), we apply a minimum-bound calibration on *t*_4_, by using a decay function from 10 Ma to ∞ (such as the offset-exponential), while the age of the root may be assigned a uniform distribution *t*_1_ ∼ U(0, 100). Ages of the non-calibration nodes (*t*_2_ and *t*_3_) have densities specified by the birth-death process. In strategy 2 (st2), we propagate the minimum and maximum bounds to all calibration nodes: the root acquires the minimum bound from node 4, while node 4 inherits the maximum age of the root, so that both nodes have joint bounds: *t*_4_ ∼ U(10, 100), and *t*_1_ ∼ U(10, 100). In strategy 3 (st3), we propagate the minimum and maximum bounds to all nodes on the phylogeny, so that *t_i_* ∼ U(10, 100) for *i* = 1, 2, 3 and 4. In all three strategies, the dating program will automatically apply a truncation so that *t*_4_ < *t*_3_ < *t*_2_ < *t*_1_. Different programs use different procedures to perform the truncation and to combine the calibration densities with the branching process model (Inoue et al., 2010). As a result the three strategies should lead to different time priors, and the different programs will also differ even for the same strategy. For simple cases, it is possible to calculate analytically the resulting marginal priors for the node ages after truncation. However, for large phylogenies with dozens of fossil calibrations, analytical calculation is impossible, and the user needs to estimate the prior by running the Bayesian MCMC program without sequence data.

Here we study how the different calibration strategies affect the time prior and the posterior time estimates. We examine two approaches used by Bayesian dating programs to combine calibration densities with the branching process to form a prior density for all node ages (the time prior): the *conditional construction* used by MCMCTree (Yang and Rannala, 2006) and the *multiplicative construction* used by BEAST (Bouckaert et al., 2014) and MrBayes (Ronquist et al., 2012b) (see Heled and Drummond, 2015). We study a simple example that is analytically tractable, and then analyze two real datasets: one of 10 primate species, and another of 48 seed plant species. We show that the different calibration strategies as well as truncation have significant impacts on the time prior and the resulting posterior time estimates. We discuss the implications of our results and give recommendations for the construction of reasonable time priors.

## 2 Material and methods

### 2.1 Fossil calibrations and the time prior

**Fig. 2:**
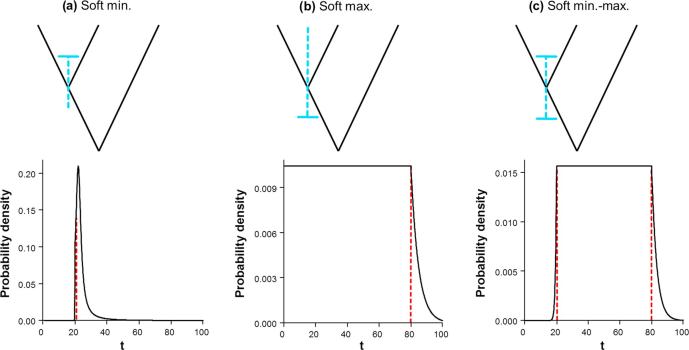
Probability densities for describing uncertainties in fossil calibrations: (a) soft minimum bound represented by a shifted-exponential distribution specified as *t_L_* = 20, *p* = 0.1, *c* = 0.1, *p_L_* = 0.01; (b) soft maximum bound specified as “*t_U_* = 80, *p_R_* = 0.05”; and (c) soft lower and upper bound, specified as “*t_L_* = 20, *t_U_* = 80, *p_L_* = 0.01, *p_U_* = 0.05”. Black solid lines represent calibration densities. Red dashed lines represent (a) minimum age (*t_L_*,), (b) maximum age (*t_U_*) and (c) both (*t_L_*, *t_U_*). (For interpretation of the references to colour in this figure legend, the reader is referred to the web version of this article.)

We consider three types of constraints on a node age based on the fossil evidence: minimum-age bound, maximum-age bound, and joint (maximum- and minimum-age) bounds (Fig.2). These are implemented in different Bayesian dating programs using different approaches.

**Minimum-age calibrations (**Fig. 2**a).** In MCMCTree, a minimum bound is represented using a truncated Cauchy distribution, denoted L(*t_L_*, *p*, *c*, *p_L_*) (Inoue et al., 2010). Here *t_L_* is the minimum age bound, *p* determines how far the mode of the distribution is from the minimum, *c* determines how sharply the distribution decays to zero, and *p_L_* is the left tail probability (i.e. the probability that the minimum bound is violated). Smaller values of *p* and *c* give a more concentrated calibration density, with a higher probability that the true age is close to the minimum age. For example, *p* = 0.1 means the mode of the distribution is at (1 + *p*)*t_L_* = 1.1*t_L_*. Here we used *p* = 0.1, *c* = 0.1, and *p_L_* = 0.01.

In MrBayes and BEAST2, minimum bounds are represented using an offset-exponential distribution (Heled and Drummond, 2012, Ronquist et al., 2012b, Bouckaert et al., 2014). If *y* has an exponential distribution with rate parameter *θ* or mean 1/*θ*, then *t* = *y* + *t_L_* has an offset-exponential distribution with parameters *θ* and *t_L_*, with mean *θ*^−1^ + *t_L_*. A large *θ* means that the true age is likely to be close to *t_L_*. In this study, we used *θ* = 10/*t_L_*, so that the mean of the distribution is 1.1 *t_L_*.

**Maximum-age calibrations (**Fig.2**b).** Maximum bounds are represented by a uniform distribution U ∼ (0, *t_U_*), where *t_U_* is the maximum age. Bounds are hard (with zero probability for any ages outside the interval) in BEAST2 and MrBayes, and soft in MCMCTree, with *p_U_* to be the error probability that the bound is violated.

**Joint (minimum- and maximum-age) calibrations (**Fig. 2**c).** Joint bounds are represented by a uniform distribution U(*t_L_*, *t_U_*) in all three programs. Again, bounds are hard in BEAST2 and MrBayes, and soft in MCMCTree, which assigns *p_L_* and *p_U_* as the error probabilities for violations of the bounds (Yang and Rannala, 2006). We use *p_L_* = 0.01 and *p_U_* = 0.05.

### 2.2 Calibration strategies to generate the time prior

The calibration strategies are different ways of generating the effective prior given the fossil bounds on the calibration nodes on the phylogeny. We consider three strategies.

**Calibration strategy st1:** Minimum and maximum constraints were applied to calibration nodes as given, without propagating onto other nodes.

**Calibration strategy st2:** Minimum and maximum constraints are propagated onto all calibration nodes, so that every calibration node has joint minimum and maximum bounds, represented by a uniform distribution. In other words, if a calibration node lacks a minimum bound, the minimum bound of its oldest descendent node is used, and if a calibration node lacks a maximum bound, the maximum bound of its youngest ancestor is used.

**Calibration strategy st3:** This is like st2 but minimum and maximum bounds are propagated onto all interior nodes on the phylogeny, so that every node has a pair of joint bounds. Note that in st2, every calibration node has a pair of bounds while in st3, every interior node has a pair of bounds.

The rooted tree topology was fixed in all analyses. This is a requirement for MCMCTree and we did the same for BEAST2 and MrBayes to avoid the confounding effects of alternative phylogenies. A constraint on the root is required in MCMCTree (Yang and Rannala, 2006) and MrBayes (Ronquist et al., 2012b). BEAST2 does not require a constraint on the root, one or more calibrations on internal nodes may be sufficient (Heled and Drummond, 2012, Heled and Drummond, 2015).

The Bayesian analysis requires a prior on the ages of all nodes on the tree. The birth-death branching process is used to provide the prior distribution for the non-calibration nodes, which is combined with the effective prior for the calibration nodes after the truncation, to generate the time prior. Two procedures have been used to achieve this in the current dating programs.

In MCMCTree, the so-called *conditional construction* is used (Yang and Rannala, 2006). Let *t_C_* be the ages of the calibration nodes, and $t_{\overset{¯}{C}}$ be the ages of the non-calibration nodes. In the example of Fig.1, *t_C_* = {*t*_1_, *t*_4_} while $t_{\overset{¯}{C}}$ = {*t*_2_, *t*_3_}. The conditional construction gives the density of all node ages as

(1) $$f(t_{C}\text{,}t_{\overset{¯}{C}})=f(t_{C})\cdot f_{\text{BDS}}(t_{\overset{¯}{C}}|t_{C})\text{,}$$

where *f*(*t_C_*) is the effective prior on the ages of the calibration nodes, given by the user-specified calibration densities after truncation, while $f_{\text{BDS}}(t_{\overset{¯}{C}}|t_{C})$ is the conditional density of the non-calibration nodes given the calibration node ages, specified by the birth-death-sampling (BDS) process (Yang and Rannala, 1997).

Both BEAST2 and MrBayes use the so-called *multiplicative construction*, in which the birth-death process density for all node ages is multiplied with the densities for the calibration nodes to generate the time prior (Heled and Drummond, 2012, Heled and Drummond, 2015).

(2) $$f(t_{C}\text{,}t_{\overset{¯}{C}})\propto f(t_{C})\cdot f_{\text{BDS}}(t_{C}\text{,}t_{\overset{¯}{C}})=f(t_{C})\cdot [f_{\text{BDS}}(t_{C})\cdot f_{\text{BDS}}(t_{\overset{¯}{C}}|t_{C})]\text{,}$$

Here *t_C_* is the density of node ages for the calibration nodes based on the user-specified calibration densities (with suitable truncation so that ancestors are older than the descendents), and *f*_BDS_(*t_C_*) is the marginal density of the node ages for the calibration nodes as specified by the birth-death-sampling process, while $f_{\text{BDS}}(t_{\overset{¯}{C}}|t_{C})$ is the conditional density of the ages of the non-calibration nodes given the ages of the calibration nodes as specified by the birth-death-sampling process. As the density of *t_C_* occurs twice in Eq. (2), this density is mathematically incorrect and “does not follow the rules of probability calculus” (Heled and Drummond, 2012). Here we treat both constructions as heuristic methods for converting user-specified constraints into the time prior.

### 2.3 Analysis of a simple example with five species

We use a simple and analytically tractable case of five species (Fig. 1) to explore the different approaches to constructing the time prior (the prior for all node ages). Nodes 1 and 4 are calibration nodes, with the fossil constraints *t*_1_ < 100 Myrs and *t*_4_ > 10, while *t*_2_ and *t*_3_ are non-calibration nodes, for which the densities are provided by a branching process such as the birth-death-sampling process. As the birth-death process has no beginning and no ending, it is necessary to condition the process either on the time of origin, or the age of the root, or on the number of sampled extant species (N) (Yang and Rannala, 1997). Here we condition on both the number of sampled extant species and the age of the root, as in Yang and Rannala (1997). Let *λ* be the per-lineage birth (speciation) rate, *μ* the per-lineage death (extinction) rate, and *ρ* the sampling fraction. We fix the parameters in the model at *λ* = *μ* = 1 and *ρ* = 0, so that the ages of the nonroot nodes are order statistics from a uniform kernel (Yang and Rannala, 1997). In other words, given the root age *t*_1_, node ages *t*_2_, *t*_3_ and *t*_4_ can be generated by sampling three independent random variables from U(0, *t*_1_) and then ordering them. The joint distribution is

(3) $$f_{\text{BDS}}(t_{4}\text{,}t_{3}\text{,}t_{2}|t_{1})=\frac{3!}{t_{1}^{3}}\text{,}0<t_{4}<t_{3}<t_{2}<t_{1}\text{.}$$

This is equivalent to the Dirichlet time prior used by Thorne et al. (1998).

**Calibration strategy 1 (st1).** We consider the *conditional* construction used by MCMCTree first (Yang and Rannala, 2006). The calibration density for *t*_1_ (the root age) is the uniform distribution

(4) $$f_{C}(t_{1})=1/t_{U}\text{,}\;0<t_{1}<t_{U}\text{,}$$

with *t_U_* = 100, while that for *t*_4_ is the offset-exponential

(5) $$f_{C}(t_{4})=\theta {\text{e}}^{-\theta (t_{4}-t_{L})}\text{,}\;t_{L}<t_{4}<\infty \text{,}$$

where *t_L_* = 10 and we choose *θ* = 1/*t_L_* so that the mean is 2*t_L_* = 20 Ma.

Multiplying those user-specified calibration densities and removing the unfeasible region (where *t*_4_ > *t*_1_) by truncation leads to the effective prior used by the dating program

(6) $$f_{C}(t_{1}\text{,}t_{4})=\frac{1}{k_{1}}\theta {\text{e}}^{-\theta (t_{4}-t_{L})}\cdot \frac{1}{t_{U}}\text{,}\;t_{L}<t_{4}<t_{1}<t_{U}\text{,}$$

where *k*_1_ = $\int_{L}^{t_{U}}\int_{t_{L}}^{t_{1}}\theta {\text{e}}^{-\theta (t_{4}-t_{L})}\cdot \frac{1}{t_{U}}\;\text{d}t_{4}\text{d}t_{1}$ = 0.80001 is a normalizing constant, to ensure that *f_C_*(*t*_1_, *t*_4_) integrates to 1.

Under the birth-death-sampling process model, with *λ* = *μ* = 1 and *ρ* = 0, the joint density for *t*_2_ and *t*_3_, conditioned on the calibration node ages (*t*_1_ and *t*_4_), is given by the fact that *t*_2_ and *t*_3_ are order statistics from U(*t*_4_, *t*_1_), with density

(7) $$f_{\text{BDS}}(t_{2}\text{,}t_{3}|t_{1}\text{,}t_{4})=2/{\left(t_{1}-t_{4}\right)}^{2}\text{,}\;t_{4}<t_{3}<t_{2}<t_{1}\text{.}$$

The effective time prior or the joint density for all node ages is thus

(8) $$f(t_{1}\text{,}t_{2}\text{,}t_{3}\text{,}t_{4})=f_{C}(t_{1}\text{,}t_{4})f_{\text{BDS}}(t_{2}\text{,}t_{3}|t_{1}\text{,}t_{4})=\frac{1}{k_{1}}\theta {\text{e}}^{-\theta (t_{4}-t_{L})}\cdot \frac{1}{t_{U}}\times \frac{2}{{\left(t_{1}-t_{4}\right)}^{2}}\text{,}\;t_{L}<t_{4}<t_{3}<t_{2}<t_{1}<t_{U}\text{,}$$

where *k*_1_ is the normalizing constant defined below equation (6).

The marginal prior densities of the calibration node ages (*t*_1_ and *t*_4_) can be obtained by integration.

(9) $$f(t_{4})=\int_{t_{4}}^{t_{U}}f_{C}(t_{1}\text{,}t_{4})\;\text{d}t_{1}=\frac{1}{k_{1}t_{U}}\theta {\text{e}}^{-\theta (t_{4}-t_{L})}\cdot (t_{U}-t_{4})\text{,}\;t_{L}<t_{4}<t_{U}\text{,}$$

(10) $$f(t_{1})=\int_{t_{L}}^{t_{1}}f_{C}(t_{1}\text{,}t_{4})\;\text{d}t_{4}=\frac{1}{k_{1}t_{U}}[1-{\text{e}}^{-\theta (t_{1}-t_{L})}]\text{,}\;t_{L}<t_{1}<t_{U}\text{,}$$

**Fig. 3:**
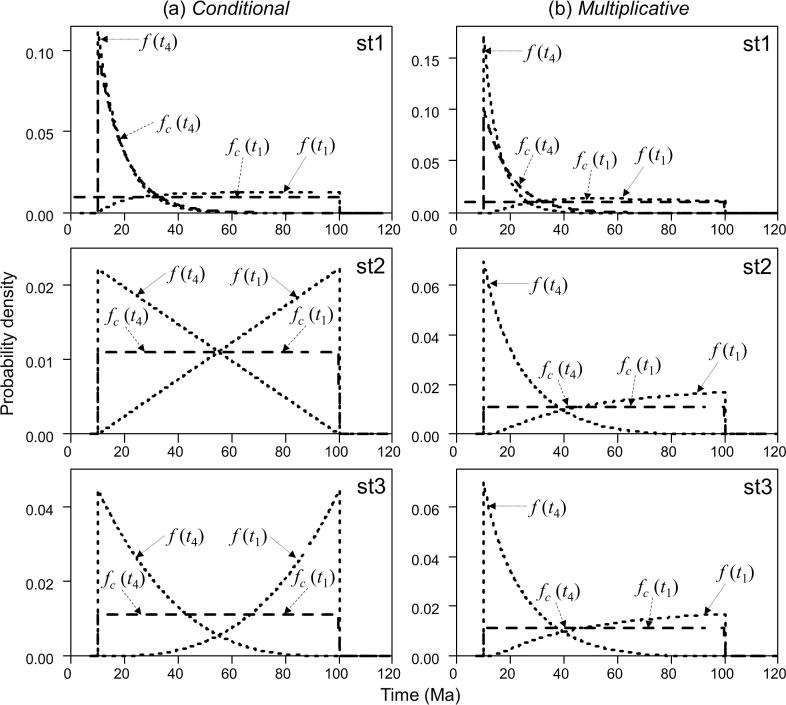
User-specified calibrations and effective priors for node ages *t*_1_ and *t*_4_ under three calibration strategies (st1, st2, st3) in a simple example of five species (Fig. 1), generated using the (a) conditional and (b) the multiplicative construction. Dashed lines represent the user-specified calibration densities, while dotted lines represent the effective prior densities.

Note that Eq. (9) can also be derived by integrating out *t*_1_, *t*_2_, *t*_3_ from *f*(*t*_1_, *t*_2_, *t*_3_, *t*_4_), and Eq. (10) can be derived by integrating out *t*_2_, *t*_3_, *t*_4_ from *f*(*t*_1_, *t*_2_, *t*_3_, *t*_4_). Fig.3a (st1) shows the user-specified calibration densities and the effective (marginal) priors after the truncation.

In the *multiplicative* construction used by BEAST and MrBayes, the densities for the calibration nodes of Eqs. (4), (5) are multiplied with the joint density of the ages of all non-root nodes from the birth-death-sampling process (Eq. (3)) to give

(11) $$\begin{array}{cc} f(t_{1}\text{,}t_{2}\text{,}t_{3}\text{,}t_{4}) & \;\neq \;\frac{1}{k_{2}}\times f_{C}(t_{1})\cdot f_{C}(t_{4})\cdot f_{\text{BDS}}(t_{2}\text{,}t_{3}\text{,}t_{4}|t_{1})\\ & =\frac{1}{k_{2}}\times \frac{1}{t_{U}}\cdot \theta {\text{e}}^{-\theta (t_{4}-t_{L})}\cdot \frac{3!}{t_{1}^{3}}\text{,}\;t_{L}<t_{4}<t_{3}<t_{2}<t_{1}\text{,} \end{array}$$

where *k*_2_ = $\int_{t_{L}}^{t_{U}}\int_{t_{L}}^{t_{1}}\int_{t_{L}}^{t_{2}}\int_{t_{L}}^{t_{3}}\frac{1}{t_{U}}\cdot \theta {\text{e}}^{-\theta (t_{4}-t_{L})}\cdot \frac{3!}{t_{1}^{3}}\;\text{d}t_{4}\text{d}t_{3}\text{d}t_{2}\text{d}t_{1}$ = 0.0174371 is a normalizing constant. Note that Eq. (11) does not make mathematical sense as two different densities occur for *t*_4_, one in *f_C_*(*t*_4_) and the other in *f*_BDS_(*t*_2_, *t*_3_, *t*_4_|*t*_1_). The marginal (effective) priors for the calibration node ages (*t*_1_ and *t*_4_) can be obtained by integration

(12) $$\begin{array}{c} f(t_{1})=\int_{t_{L}}^{t_{1}}\int_{t_{L}}^{t_{2}}\int_{t_{L}}^{t_{3}}f(t_{1}\text{,}t_{2}\text{,}t_{3}\text{,}t_{4})\;\text{d}t_{4}\text{d}t_{3}\text{d}t_{2}=\frac{3!}{k_{2}t_{U}t_{1}^{3}}\left[\frac{{\left(t_{1}-t_{L}\right)}^{2}}{2}+\frac{{\left(t_{L}-t_{1}\right)}^{2}}{\theta }+\frac{1-e^{-\theta (t_{1}-t_{L})}}{\theta^{2}}\right]\text{,}\\ f(t_{4})=\int_{t_{4}}^{t_{U}}\int_{t_{3}}^{t_{U}}\int_{t_{2}}^{t_{U}}f(t_{1}\text{,}t_{2}\text{,}t_{3}\text{,}t_{4})\;\text{d}t_{1}\text{d}t_{2}\text{d}t_{3}=\frac{3\theta e^{-\theta (t_{4}-t_{L})}}{k_{2}t_{U}}\left[\log\frac{t_{U}}{t_{4}}-1.5+\frac{2t_{4}}{t_{U}}-\frac{t_{4}^{2}}{2t_{U}^{2}}\right]\text{,} \end{array}$$

with *t_L_* < *t*_1_ < *t_U_* and *t_L_* < *t*_4_ < *t_U_*. Fig.3b (st1) shows the user-specified calibration densities and the effective (marginal) priors after the truncation.

**Calibration strategy 2 (st2).** The minimum and maximum bounds are propagated onto all calibration nodes so that the calibration densities are

(13) $$\begin{array}{cc} f_{C}(t_{1})=1/(t_{U}-t_{L})\text{,} & t_{L}<t_{1}<t_{U}\text{,}\\ f_{C}(t_{4})=1/(t_{U}-t_{L})\text{,} & t_{L}<t_{4}<t_{U}\text{.} \end{array}$$

We first consider the *conditional* construction. After truncation, the effective joint prior for *t*_1_ and *t*_4_ becomes, in contrast to Eq. (6),

(14) $$f_{C}(t_{1}\text{,}t_{4})=2/{\left(t_{U}-t_{L}\right)}^{2}\text{,}\;t_{L}<t_{4}<t_{1}<t_{U}\text{.}$$

This is multiplied with the birth-death-sampling process density for the non-calibration nodes of Eq. (7) to give the time prior as

(15) $$f(t_{1}\text{,}t_{2}\text{,}t_{3}\text{,}t_{4})=f_{C}(t_{1}\text{,}t_{4})\cdot f_{\text{BDS}}(t_{2}\text{,}t_{3}|t_{1}\text{,}t_{4})=\frac{2}{{\left(t_{U}-t_{L}\right)}^{2}}\times \frac{2}{{\left(t_{1}-t_{4}\right)}^{2}}\text{,}\;t_{L}<t_{4}<t_{3}<t_{2}<t_{1}<t_{U}\text{.}$$

The marginal densities for the calibration node ages are

(16) $$\begin{array}{cc} f(t_{1})=\int_{t_{L}}^{t_{1}}f_{C}(t_{1}\text{,}t_{4})\;\text{d}t_{4}=\frac{2}{{\left(t_{U}-t_{L}\right)}^{2}}\times (t_{1}-t_{L})\text{,} & t_{L}<t_{1}<t_{U}\text{,}\\ f(t_{4})=\int_{t_{4}}^{t_{U}}f_{C}(t_{1}\text{,}t_{4})\;\text{d}t_{1}=\frac{2}{{\left(t_{U}-t_{L}\right)}^{2}}\times (t_{U}-t_{4})\text{,} & t_{L}<t_{4}<t_{U}\text{.} \end{array}$$

Fig.3a (st2) shows the densities.

With the *multiplicative* construction, the time prior is given by multiplying the calibration densities (Eq. (13)) with the birth-death-sampling density for the noncalibration nodes (Eq. (3)) and then applying truncation

(17) $$f(t_{1}\text{,}t_{2}\text{,}t_{3}\text{,}t_{4})=\frac{1}{k_{3}}\times \frac{1}{{\left(t_{U}-t_{L}\right)}^{2}}\cdot \frac{3!}{t_{1}^{3}}\text{,}t_{L}<t_{4}<t_{3}<t_{2}<t_{1}<t_{U}\text{,}$$

where *k*_3_ = $\int_{t_{L}}^{t_{U}}\int_{t_{L}}^{t_{1}}\int_{t_{L}}^{t_{2}}\int_{t_{L}}^{t_{3}}\frac{1}{{\left(t_{U}-t_{L}\right)}^{2}}\cdot \frac{3!}{t_{1}^{3}}\;\text{d}t_{4}\text{d}t_{3}\text{d}t_{2}\text{d}t_{1}$ = 0.00530524 is a normalizing constant, calculated numerically. The marginal (effective) priors for the calibration nodes (*t*_1_ and *t*_4_) are then

(18) $$\begin{array}{c} f(t_{1})=\int_{t_{L}}^{t_{1}}\int_{t_{L}}^{t_{2}}\int_{t_{L}}^{t_{3}}f(t_{1}\text{,}t_{2}\text{,}t_{3}\text{,}t_{4})\;\text{d}t_{4}\text{d}t_{3}\text{d}t_{2}=\frac{3}{k_{3}{\left(t_{U}-t_{L}\right)}^{2}}\left[\log\frac{t_{U}}{t_{4}}-1.5+\frac{2t_{4}}{t_{U}}-\frac{t_{4}^{2}}{2t_{U}^{2}}\right]\text{,}\\ f(t_{4})=\int_{t_{4}}^{t_{U}}\int_{t_{3}}^{t_{U}}\int_{t_{2}}^{t_{U}}f(t_{1}\text{,}t_{2}\text{,}t_{3}\text{,}t_{4})\;\text{d}t_{1}\text{d}t_{2}\text{d}t_{3}=\frac{{\left(t_{1}-t_{L}\right)}^{3}}{k_{3}t_{1}^{3}{\left(t_{U}-t_{L}\right)}^{2}}\text{,} \end{array}$$

with *t_L_* < *t*_1_ < *t_U_* and *t_L_* < *t*_4_ < *t_U_*. Fig.3b (st2) shows the user-specified calibration densities and the effective (marginal) priors after the truncation.

**Calibration strategy 3 (st3).** The minimum and maximum bounds are propagated onto all nodes on the phylogeny, so that every node has joint bounds: *f_C_*(*t_i_*) = 1/(*t_U_* − *t_L_*), *t_L_* < *t_i_* < *t_U_*, for *i* = 1, 2, 3, 4. With the *conditional* construction, the birth-death-sampling model plays no role in the construction of the time prior since all nodes have calibration information. After truncation, the effective time prior is

(19) $$f(t_{1}\text{,}t_{2}\text{,}t_{3}\text{,}t_{4})=\frac{4!}{{\left(t_{U}-t_{L}\right)}^{4}}\text{,}t_{L}<t_{4}<t_{3}<t_{2}<t_{1}<t_{U}\text{.}$$

Since *t*_4_ is the smallest of four independent and identically distributed (i.i.d.) random variables and *t*_1_ is the largest, their marginal densities are given by the distribution of order statistics

(20) $$\begin{array}{cc} f(t_{1})=4\cdot {\left(\frac{t_{1}-t_{L}}{t_{U}-t_{L}}\right)}^{3}\cdot \frac{1}{t_{U}-t_{L}}\text{,} & t_{L}<t_{1}<t_{U}\text{,}\\ f(t_{4})=4\cdot {\left(\frac{t_{U}-t_{4}}{t_{U}-t_{L}}\right)}^{3}\cdot \frac{1}{t_{U}-t_{L}}\text{,} & t_{L}<t_{4}<t_{U}\text{.} \end{array}$$

Fig.3a (st3) shows the densities. Truncation now has a strong effect.

With the *multiplicative* construction, two options seem possible. The first is to ignore the birth-death process density since all the node ages have calibration with this strategy. This is then equivalent to the conditional construction of MCMCTree. The second is to multiply the calibration densities (Eq. (19)) with the birth-death-sampling density of Eq. (3), followed by a truncation to give

(21) $$f(t_{1}\text{,}t_{2}\text{,}t_{3}\text{,}t_{4})=\frac{1}{k_{4}}\frac{4!}{{\left(t_{U}-t_{L}\right)}^{4}}\times \frac{3!}{t_{1}^{3}}\text{,}\;t_{L}<t_{4}<t_{3}<t_{2}<t_{1}<t_{U}\text{,}$$

where the normalizing constant *k*_4_ = $\int_{t_{L}}^{t_{U}}\int_{t_{L}}^{t_{1}}\int_{t_{L}}^{t_{2}}\int_{t_{L}}^{t_{3}}\frac{4!}{{\left(t_{U}-t_{L}\right)}^{4}}\times \frac{3!}{t_{1}^{3}}\;\text{d}t_{4}\text{d}t_{3}\text{d}t_{2}\text{d}t_{1}$ = 0.000015719. The marginal priors for *t*_1_ and *t*_4_ are then

(22) $$\begin{array}{c} f(t_{1})=\int_{t_{L}}^{t_{1}}\int_{t_{L}}^{t_{2}}\int_{t_{L}}^{t_{3}}f(t_{1}\text{,}t_{2}\text{,}t_{3}\text{,}t_{4})\;\text{d}t_{4}\text{d}t_{3}\text{d}t_{2}=\frac{4!\times 3}{k_{4}{\left(t_{U}-t_{L}\right)}^{4}}\left[\log\frac{t_{U}}{t_{4}}-1.5+\frac{2t_{4}}{t_{U}}-\frac{2t_{4}^{2}}{2t_{U}^{2}}\right]\text{,}\\ f(t_{4})=\int_{t_{4}}^{t_{U}}\int_{t_{3}}^{t_{U}}\int_{t_{2}}^{t_{U}}f(t_{1}\text{,}t_{2}\text{,}t_{3}\text{,}t_{4})\;\text{d}t_{1}\text{d}t_{2}\text{d}t_{3}=\frac{4!\times {\left(t_{1}-t_{L}\right)}^{3}}{k_{4}t_{1}^{3}{\left(t_{U}-t_{L}\right)}^{4}}\text{,} \end{array}$$

with *t_L_* < *t*_1_ < *t_U_* and *t_L_* < *t*_4_ < *t_U_*. Fig.3b (st3) shows the user-specified calibration densities and the effective (marginal) priors after the truncation.

The calibration densities and the effective time priors generated by the conditional and the multiplicative constructions using the three calibration strategies are plotted in Fig.3. From Fig.3a it is apparent that with the conditional construction strategy st1 generates marginal priors that are closest to the original calibration densities. This is because the youngest node is calibrated with an offset-exponential distribution with a relatively short tail, and so truncation between the two calibration densities is minimal. In Strategy st2 the youngest node inherits the maximum age constraint from the root. This strategy avoids the choice of arbitrary parameters in the Cauchy or shifted-exponential calibrations. In this case truncation is more severe, and the marginal prior densities differ substantially from the calibration densities. In strategy st3, the inclusion of two additional calibration densities for *t*_3_ and *t*_2_ increases the truncation effect, and the result is that the marginal priors on *t*_4_ and *t*_1_ are pushed apart. The multiplicative construction is shown in Fig.3b. Strategy st1 generates marginal priors that are closest to the original calibration densities, while truncation has a major impact in strategies st2 and st3, so that the marginal prior densities differ substantially from the calibration. St2 and st3 generate nearly identical prior densities. Overall Fig.3 shows that the conditional and the multiplicative constructions, as well as the different calibration strategies, generate very different effective time priors.

### 2.4 Analysis of the primate dataset

**Fig. 4:**
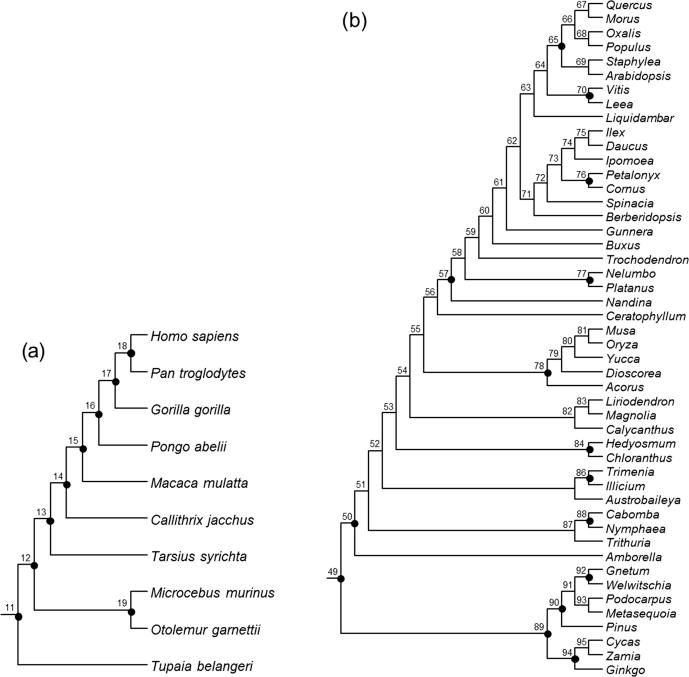
Phylogenies for (a) 10 primate species, and (b) 48 seed plant species. Calibration nodes are indicated by black solid circles.

We used eight mitochondrial coding genes (Cyt B, CO1, CO2, CO3, ND2, ND3, ND4 and ND4 L) and the mitochondrial 12S and 16S ribosomal RNA (rRNA) genes from nine primate species and an outgroup (*Tupaia belangeri*) (Fig.4a) (GenBank accession numbers in Table S1). We partitioned the data into three partitions: (1) 1st and 2nd codon positions; (2) 3rd codon positions and (3) rRNA genes. The final alignment had 9361 base pairs, with 11.1% of missing data. The data were analyzed using the three dating programs (MCMCTree, BEAST2, and MrBayes), under the independent-rates model to construct the prior of the rates. The time unit is set at 100 Myrs. The sequence likelihood was calculated under the HKY+Γ_5_ substitution model (Hasegawa et al., 1985, Yang, 1994), with separate substitution-rate parameters assigned and estimated for each partition.

There are nine fossil calibrations (Table 1) (dos Reis et al., 2012), five of which are joint minimum and maximum bounds, while the other four are minimum bounds only. We implemented calibration strategies st1 and st2 in the programs MCMCTree, BEAST2, and MrBayes. As all nine interior nodes have calibration information, st3 is equivalent to st2. Bounds are soft in MCMCTree, and hard in BEAST2 and MrBayes. Minimum bounds are implemented using the truncated Cauchy distribution in MCMCTree and the offset-exponential distribution in BEAST2 and MrBayes.

**Table 1:** Primate fossil calibrations used in this study.

Node	Clade	Minimum (Ma)	Maximum (Ma)
11	Scandentia-Primates	61.5 (†*Carpolestidae*)	130 (absence of placentals)
12	Primates (*Otolemur*-Human)	55.6 (†*Altiatlasius*)	–
13	Haplorhini (*Tarsius*-Human)	45 (†*Tarsius*)	–
14	Anthropoidea (*Callithrix*-Human)	33.7 (†*Catopithecus*)	–
15	Catarrhini (*Macaca*-Human)	23.5 (†*Proconsul*)	34 (absence of hominoids)
16	Hominidae (*Pongo*-Human)	11.2 (†*Sivapithecus*)	33.7 (absence of pongines)
17	Ponginae (*Gorilla-Pan*/Human)	7.25 (†*Chororapithecus*)	–
18	Homininae (*Pan*-Human)	5.7 (†*Orrorin*)	10 (absence of hominines)
19	Lorisoidea (*Otolemur- Microcebeus*)	33.7(†*Karanisia*)	55.6 (absence of strepsirrhines)

Note: All calibrations are derived from dos Reis et al. (2012). Fossil taxa are indicated by a dagger (†) before their names.

In MCMCTree, the parameters of the birth-death-sampling process are fixed at *λ* = *μ* = 1, and *ρ* = 0. These specify a uniform kernel. The independent-rates model (IR) assumes that the rates for branches are independent variables from the lognormal distribution, specified by the mean of the rate (*μ*) and the variance of the log rate *σ*^2^ (which determines the extent of rate variation across branches) (Rannala and Yang, 2007). The mean rate is assigned a gamma hyperprior G(2, 2) with mean 2/2 = 1.0 substitutions per site per time unit (100MY) or 10^−8^ substitutions per site per year, and the rate drift parameter is assigned another gamma hyperprior, *σ*^2^ ∼ G(1, 10), with mean 0.1.

Both BEAST2 and MrBayes assign hyperpriors to implement the birth-death-sampling model: the net diversification rate *λ* − *μ* ∼ U(0, 1) and the relative extinction rate *μ*/*λ* ∼ U(0, 1) (Stadler, 2010, Hohna et al., 2011). In MrBayes the sampling probability (*ρ*) is fixed at 0.02.

In BEAST2 we specified a *Relaxed Clock Log Normal* (ucld) model, which assumes that the substitution rates for branches are independent variables from a lognormal distribution (Drummond et al., 2006). The lognormal distribution is parametrized using the mean and the standard deviation. The mean (*ucldMean.c*) was assigned a gamma hyperprior G(2, 0.5) with mean 1.0, and the standard deviation (*ucldStdev.c*) was assigned a gamma hyperprior G(2, 0.05) with mean 0.1.

In MrBayes we used the *Independent Gamma Rate* (IGR) model in where the rates for branches are independent variables from a gamma distribution (Lepage et al., 2007). The gamma model is parametrized using two parameters: the mean and variance. The mean is assigned a lognormal hyperprior LN(−0.125, 0.5), with the mean exp{−0.125 + 0.5^2^/2} = 1.0. The variance (*Igrvarpr*) is assigned an exponential prior with mean 0.1.

The MCMC sampling settings were determined through pilot runs and differed among the programs. We ran each program at least twice, and checked for convergence by comparing the posterior mean estimates between runs and by plotting the time series traces of the samples. We then merged the samples from the runs before summarizing the posterior. For MCMCTree, two runs were performed, each consisting of 2 × 10^6^ iterations after a burn-in of 4 × 10^4^ iterations and sampling every 200, resulting in a total of 2 × 10^4^ samples from the two runs. For MrBayes, two runs were performed, each consisting of 2 × 10^6^ iterations, sampling every 100, with the burn-in set to 25% of samples, resulting in a total of 3 × 10^4^ samples from the two runs. For BEAST2 we performed three runs, each consisting of 10^7^ iterations, sampling every 1000. The burn-in was set to 30% of samples, resulting in a total of 21,000 samples from all three runs.

### 2.5 Analysis of the seed plant dataset

We used five plastid genes (*atpB, matK, NdhF, rbcL*, and *rps4*) and two nuclear RNA genes (*18s* and *26s*) for 48 seed plant species (GenBank accession numbers in Table S2) from Barba-Montoya et al. (submitted for publication). The tree topology of Fig.4b is fixed. The sequence alignment had 13,211 base pairs, with 26% missing data. We treated the data as three partitions: (1) 1st and 2nd codon positions for plastid genes; (2) 3rd positions for plastid genes and (3) nuclear RNA genes. The data were analyzed using the three programs (MCMCTree, BEAST2, and MrBayes), with similar settings as in the analysis of the primate dataset, but some modifications were necessary to accommodate the differences in the time scale and in the rate. The sequence likelihood was calculated under the HKY+Γ_5_ substitution model (Hasegawa et al., 1985, Yang, 1994), with separate substitution-rate parameters assigned and estimated for each partition. In MCMCTree the approximate likelihood method (Thorne et al., 1998, dos Reis and Yang, 2011) is used to calculate the sequence likelihood, using the maximum likelihood estimates of branch lengths and the Hessian matrix. In BEAST2 and MrBayes the sequence likelihood was calculated exactly.

There are 15 fossil calibrations on the tree (Fig.4b) (Barba-Montoya et al., submitted for publication) Among them seven are joint minimum and maximum bounds and eight are minimum bounds (Table 2). The time unit is set to 100 Myrs. The calibration information is implemented in the three programs using the three strategies as described earlier.

**Table 2:** Seed plant fossil calibrations used in this study.

Node	Clade	Minimum divergence time (Ma)	Maximum divergence time (Ma)
49	Spermatophytes (*Ginkgo-Quercus*)	308.14 (†*Cordaites iowensis*)	365.63 (base of Vco zone which contains the first seeds)
50	Angiosperms (*Amborella-Quercus*)	125.9 (tricolpate pollen)	247.3 (sediments below the oldest occurrence of angiosperm like pollen which are devoid of such pollen)
57	Eudicots without *Ceratophyllum* (*Nandina-Quercus)*	119.6 (†*Hyrcantha decussata*)	–
65*	No name *(Arabidopsis-Quercus)*	82.8 (†*Paleoclusia chevalieri* and †*Dressiantha bicarpellata*)	127.2 (oldest potential age of tricolpate pollen)
70	Vitales (*Vitis-Leea*)	65.6 (†*Indovitis chitaleyae)*	–
76	Cornales (*Petalonix-Cornus*)	85.8 (†*Tylerianthus crossmanensis*)	–
77	Proteales (*Nelumbo-Platanus*)	107.59 (†*Sapindopsis variabilis*, †*Aquia brookensis* and †*Palatonocarpus brookensis*)	–
78	Monocots (*Acorus-Musa*)	112.6 (†*Liliacidites*)	–
84	Chloranthales (*Chloranthus-Hedyosmum*)	92.8 (†*Pennipolis*)	–
86	No name (*Trimenia-Illicium*)	107.59 (†*Anacostia virginiensis*)	–
88	Cabombaceae (*Cabomba-Nymphaea*)	111 (†*Pluricarpellatia peltata*)	–
89	Acrogymnospermae (*Ginkgo-Pinus*)	308.14 (†*Cordaties iowensis*)	365.7 (base of Vco zone which contains the first seeds)
90	Conifers (*Pinus-Metasequoia*)	147 (†*Rissikia media*)	312.38 (sediments bearing †*Cordaites iowensis*)
92	Gnetales (*Gnetum-Welwitschia*)	119.6 (†*Eoantha zherkihinii*)	312.38 (sediments bearing †*Cordaites iowensis*)
94	No name (*Ginkgo-Cycas*)	264.7 (†*Crossozamia*)	365.63 (base of Vco zone which contains the first seeds)

Note: Calibrations are derived from Barba-Montoya et al. (submitted for publication) and (*) from Clarke et al. (2011). Fossil taxa are indicated by a dagger (†) before their names.

In MCMCTree, the parameters of the birth-death-sampling process are fixed at *λ* = *μ* = 1, and *ρ* = 0. We used the independent-rates (IR) model, with the overall rate assigned a gamma hyperprior G(2, 30) with mean 2/30 = 0.067 substitutions per site per 100MY, and with the variance of the log-rate assigned a gamma hyperprior *σ*^2^ ∼ G(2, 20) with mean 0.1. Two runs were performed, each consisting of 10^6^ iterations after a burn-in of 40,000 iterations and sampling every 200. The combined sample of 10,000 samples was used to summarize.

In the BEAST2 and MrBayes analyses, hyperpriors are assigned to parameters in the birth-death-sampling model: *λ* − *μ* ∼ U(0, 1) and *μ*/*λ* ∼ U(0, 1) (Stadler, 2010, Hohna et al., 2011). In MrBayes, the sampling probability (*ρ*) is fixed at 0.0002.

In BEAST2 we specified the ucld model. The mean of the lognormal (*ucldMean.c*) was assigned a gamma hyperprior G(2, 0.0335) with mean 0.067, and the standard deviation of the lognormal (*ucldStdev.c*) was assigned a gamma hyperprior G(2, 0.05) with mean 0.1. Three runs were performed, each consisting of 10^7^ iterations, sampling every 1000. The burn-in was set to 30% of samples, resulting in a total of 21,000 samples from the posterior from the three runs.

In MrBayes we used the *Independent Gamma Rate* (IGR) model. The mean of the gamma was assigned a lognormal hyperprior LN(−2.79, 0.5^2^), with the mean exp{−2.79 + 0.5^2^/2} = 0.07, and the variance of the gamma is assigned an exponential hyperprior with mean 0.1. Four runs were performed, each consisting of 1.5 × 10^6^ iterations, sampling every 100. The burn-in was set to 33.3% of samples, resulting in a total of 4 × 10^4^ samples from all four runs.

## 3 Results

### 3.1 Analysis of a simple example with five species

**Fig. 5:**
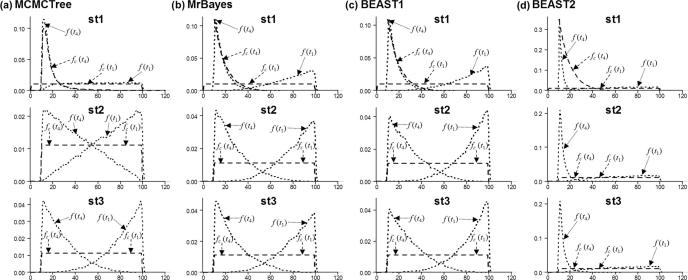
User-specified calibrations and effective priors for node ages *t*_1_ and *t*_4_ under three calibration strategies (st1, st2, st3) in a simple example of five species (Fig. . 1), generated using (a) MCMCTree; (b) MrBayes; (c) BEAST1 and (d) BEAST2. Dashed lines represent the user-specified calibration densities, while dotted lines represent the effective prior densities.

Fig. 5 shows the results from analysing this example using the three different dating programs. In MCMCTree (Fig. 5a) the calibration density used for *t*_4_ in strategy st1, is the Cauchy distribution (shifted-exponential) with parameters *t_L_* = 10, *p* = 0.2, *c* = 0.5 and *p_L_* = 0.0001. We fix the parameters in the birth-death-sampling model at *λ* = *μ* = 1 and *ρ* = 0 in all strategies. The prior densities generated by the three calibration strategies using MCMCTree (Fig. 5a, st1, st2, st3) are almost identical to those from the conditional construction (Fig. 3a, st1, st2, st3).

To examine the implementation in MrBayes and BEAST (Fig. s5b–d) we fix the parameters in the birth-death-sampling model at *λ* = μ = 1 and *ρ* = 0. To avoid numerical problems, we used *λ* = 1.001, *μ* = 0.999 and *ρ* = 0.0001. In MrBayes the net diversification rate *λ* − *μ* is fixed at 0.002, the relative extinction rate *μ*/*λ* is fixed at 0.998 and the sampling probability (*ρ*) is fixed at 0.0001. In BEAST1 and BEAST2 we use for the net diversification rate *λ* − *μ* a uniform distribution U(0.00199, 0.00201) and for the relative extinction rate *μ*/*λ* U(0.99799, 0.99801). In BEAST1 we use U(0.000099, 0.000101) for the sampling probability (*ρ*). None of these programs generated identical results to the multiplicative construction. The prior densities generated by MrBayes and BEAST1 were similar but not identical. Precise reasons for the discrepancies between the analytical example, BEAST1 and MrBayes are unknown. One possible reason is that BEAST1 and MrBayes may not be conditioning the birth-death-sampling age density on both root (*t*_1_) and the number of sampled species (*N*). Here we emphasize the large differences in the prior generated by the conditional and multiplicative constructions and the priors from the three calibration strategies.

### 3.2 Analysis of the primate dataset

**Fig. 6:**
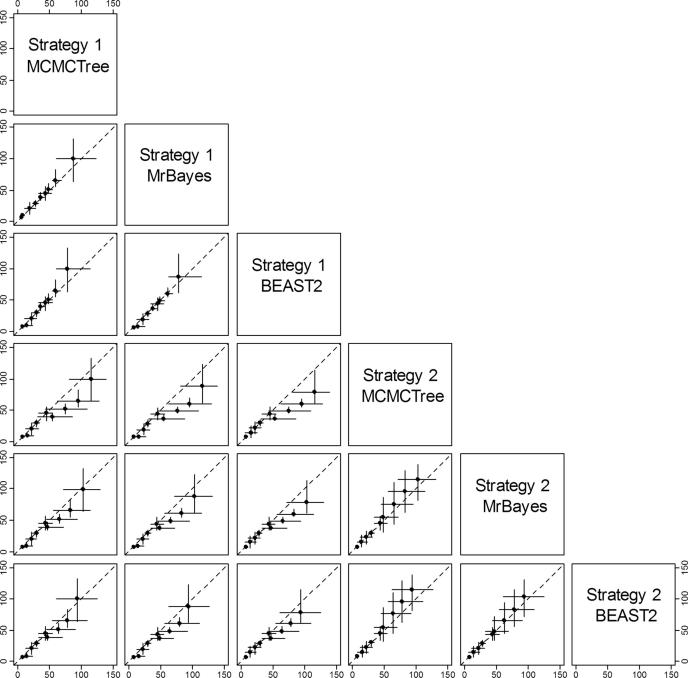
Means and 95% CIs in the time prior (the prior for node ages) on the primate phylogeny (Fig. 5a) generated using calibration strategies st1 and st2 and three dating programs: MCMCTree, BEAST2 and MrBayes.

**Fig. 7:**
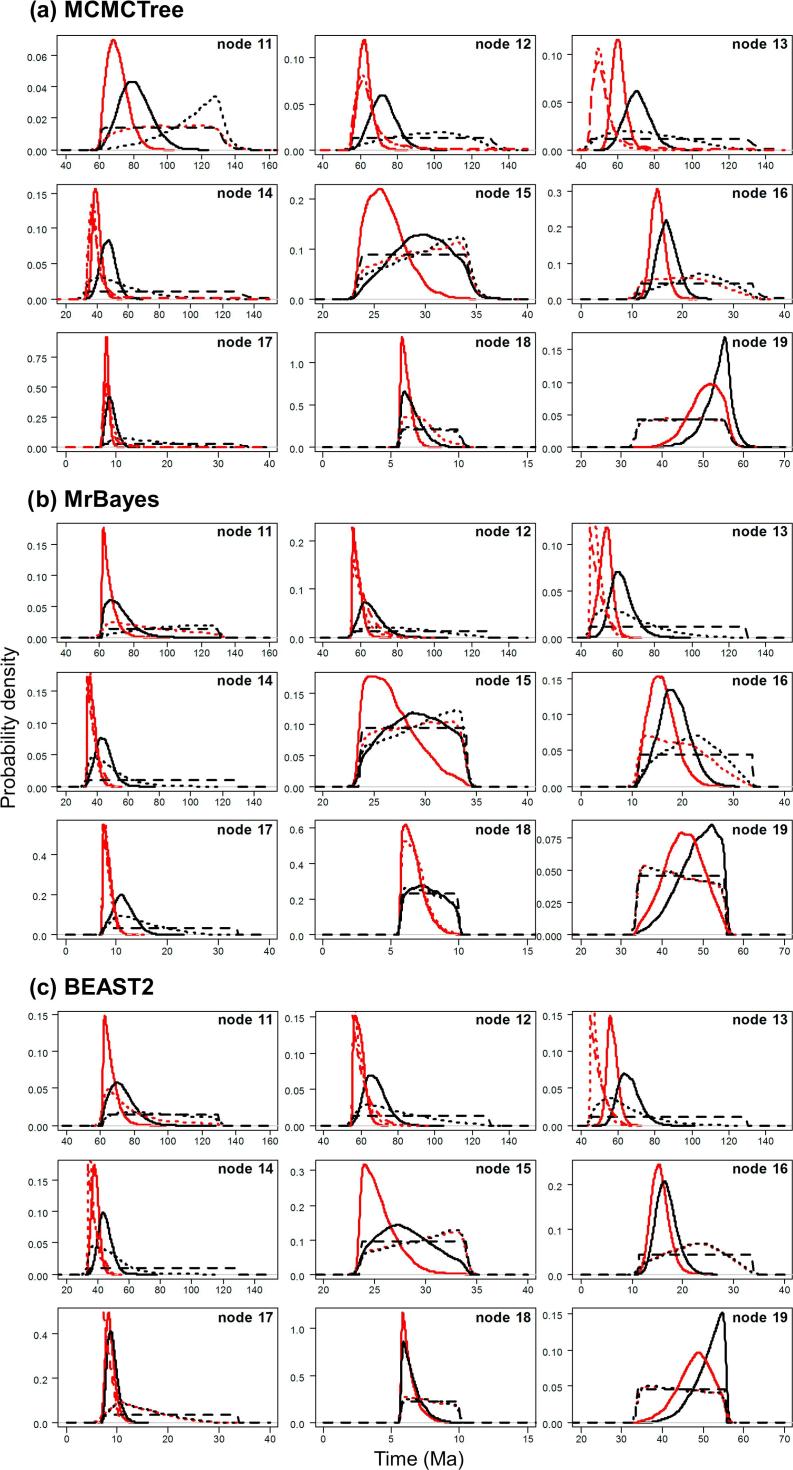
User-specified calibration densities (dashed lines), effective time priors (dotted lines), and the posterior (solid lines) for the primate dataset, under calibration strategies st1 (red) and st2 (black), implemented in MCMCTree, BEAST2 and MrBayes.

**Fig. 8:**
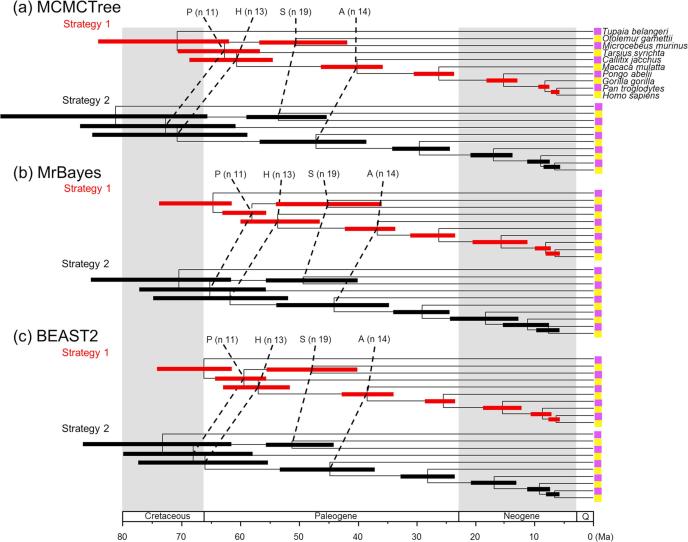
Timetrees showing posterior divergence time estimates for the primates. The branches are drawn to reflect the posterior means of node ages and the bars represent 95% HPD intervals. The dataset was analysed using MCMCTree, MrBayes amd BEAST2 under the independent-rates model, using calibration strategies st1 and st2.

The calibration densities and the effective time priors generated by the three programs using calibration strategies st1 and st2 are plotted in Fig. 6, Fig. 7. The posterior distributions of divergence times are shown in Fig. 7, Fig. 8.

First, we note that with both st1 and st2, the user-specified calibration densities are very different from the marginal densities for the node ages in the effective time prior. This difference is mainly caused by the truncation to enforce the constraint that ancestors are older than descendants. In particular, the root age assigned a pair of bounds represented by the uniform distribution, and in the time prior, the density is pushed towards the maximum. Node 18 is a descendent to many other interior nodes but is ancestral to none, so that its density is pushed towards the minimum. The patterns for other nodes are more complex. Second, strategy st2, which uses uniform bounds for all interior nodes, show much greater truncation effect so that the user-specified calibration densities and the marginal prior densities are even more different than under strategy st1. Third, the differences in the prior of node ages are transferred to the differences in the posterior. For example, the prior favoured much older age for the root under st2 than under st1 for all three programs (Fig.7a–c, node 11), and this pattern persisted in the posterior.

Lastly, the three dating programs produced similar priors and posteriors (Fig. 7, Fig. 8), although MCMCTree produced slightly older time estimates and wider intervals, especially for old nodes such as the root.

### 3.3 Analysis of seed plant dataset

**Fig. 9:**
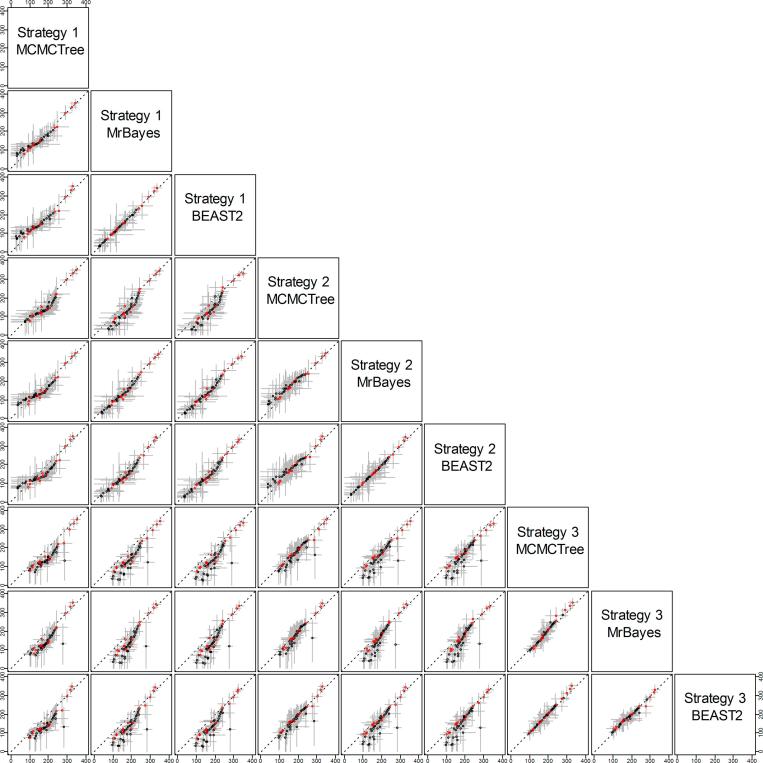
Means and 95% CIs in the time prior for node ages on the seed plant phylogeny (Fig. 5b) generated using three calibration strategies (st1-3) and three dating programs: MCMCTree, BEAST2 and MrBayes. Calibration nodes are highlighted in red.

**Fig. 10:**
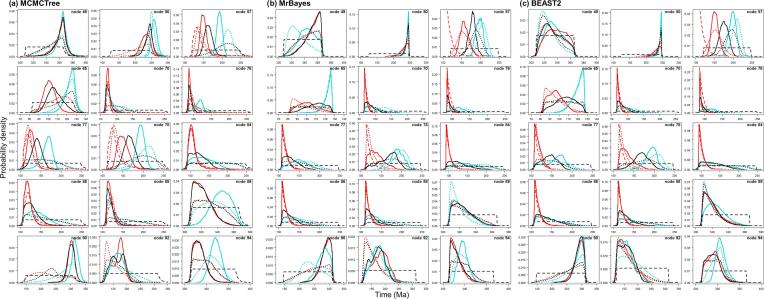
User-specified calibration densities (dashed lines), effective time priors (dotted lines), and the posterior (solid lines) for the seed plant dataset, under calibration strategies st1 (red), st2 (black), and st3 (blue), implemented in MCMCTree, BEAST2 and MrBayes. Only the 15 calibration nodes are used in the plots.

**Fig. 11:**
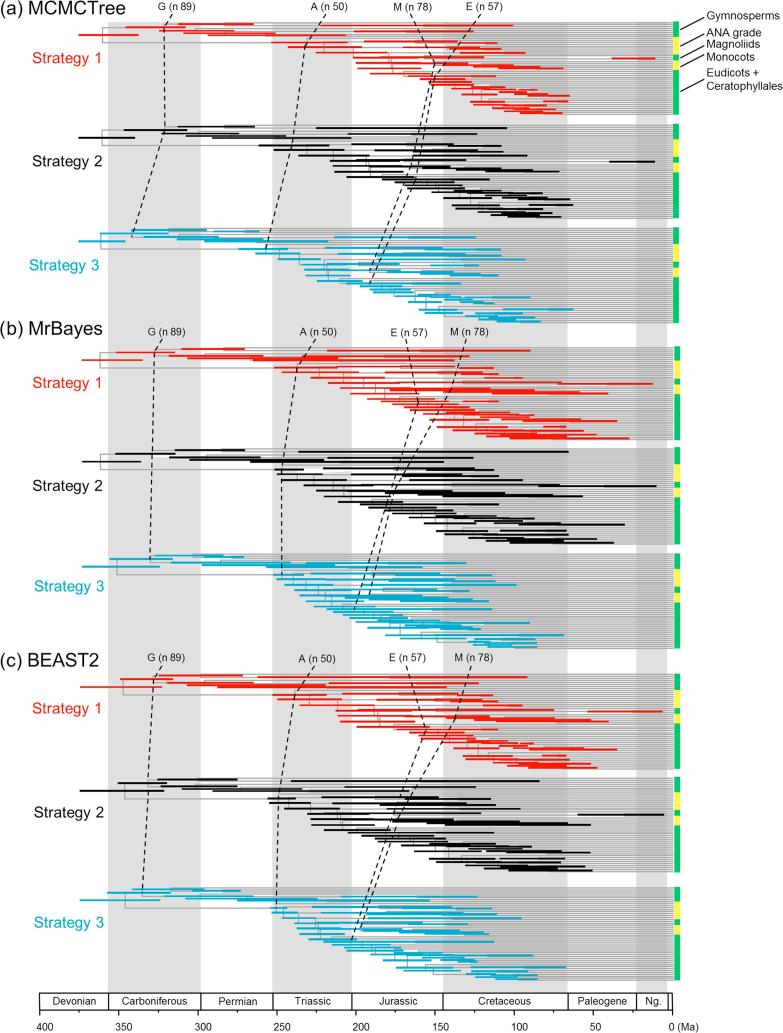
Timetrees showing posterior divergence time estimates for major seed plant groups. The branches are drawn to reflect the posterior means of node ages and the bars represent 95% HPD intervals. The dataset was analysed using MCMCTree, MrBayes amd BEAST2 under the independent-rates model, using three calibration strategies: st1, st2, and st3.

The calibration densities and the effective time priors generated by the three programs using the three calibration strategies are plotted in Fig. 9, Fig. 10. The posterior distributions of node ages are shown in Fig. 10, Fig. 11. We see similar patterns to those in the analysis of the primate dataset. First, there are large differences between calibration densities specified by the user on one hand and the (marginal) effective prior densities used by the dating software on the other. The difference is particularly pronounced for nodes with wide joint bounds as the effective prior used by the dating software is much narrower. Furthermore, truncation pushes the ages of old nodes such as the root towards the user-specified maximum bound, or even outside the maximum bound in the case of MCMCTree, which allows bound violation due to its use of soft bounds (e.g., Fig.10a–c, node 49). At the same time, truncation has the effect of pushing the ages of younger nodes towards the minimum bound in the prior (e.g., Fig.10a–c, nodes 86, 88, and 89).

Second, as in the case of the primate dataset, the posterior of the node ages is sensitive to the prior, and differences in the time prior are directly transferred to differences in the posterior. For example, nodes 77 and 78 are older under st2 than under st1 and even older under st3, and exactly the same trend persists in the posterior (Fig.10a–c). This pattern holds for all three dating programs.

Third, strategies st2 and st3 showed greater truncation effects so that the user-specified calibration densities and the marginal prior densities are even more different than under st1. The large differences in the priors of the three strategies persisted in the posterior. The time estimates tended to be older under st2 than under st1, while st3 produced the oldest time estimates (Fig. 10, Fig. 11). For example, the posterior mean estimated using st1 suggests that the eudicots (node 57) originated around 155 Ma, but using st3 the posterior mean was around 195 Ma, with a difference of 40 Myrs. The origin of monocots (node 78) was dated to ∼136 Ma under st1 in BEAST2 and MrBayes and 150 Ma in MCMCTree, but using st3 the posterior mean for this node was around 190 Ma, with again a difference of ∼40 Myrs. These differences in the posterior reflect the differences in the time prior generated under the three strategies (Fig. 10, Fig. 11).

Differences in posterior time estimates exist among the three dating programs, reflecting their different procedures to construct the time prior using the same fossil-calibration information (Fig. 9, Fig. 10). BEAST2 produced slightly younger estimates of root age (node 49) and MCMCTree produced narrower intervals than BEAST2 and MrBayes. The differences among the dating programs in both the prior and the posterior are the smallest for calibration strategy st3. This is because with st3 all nodes on the phylogeny were calibrated, so that the birth-death-sampling process plays no or little role in specifying the time prior.

## 4 Discussion

In a conventional Bayesian analysis, the posterior distribution of the parameters converge to a point mass (the true value of the parameter) and the prior becomes less and less important when the amount of data approaches infinity. Bayesian molecular clock dating is an unconventional estimation problem in the sense that such convergence to truth does not occur (Yang and Rannala, 2006). If the amount of molecular data increases and the fossil calibration information is fixed, the posterior will not converge to a point or to the true node ages, and furthermore the prior will continue to exert a large impact on the posterior. Even if we use whole genomes in the dating analysis so that sequence distances and branch lengths are estimated with virtually no random sampling errors, fossil calibrations and the time prior constructed using the fossil calibrations will remain important to the posterior time estimates. The fundamental difficulty faced by the dating analysis is the confounding effect of time and rate in sequence comparisons: molecular data provide information about the genetic distances, and only fossil calibrations (or dated geological events) can resolve the distances into absolute times and absolute rates. The asymptotic dynamics of the dating problem has been characterized in the infinite-sites theory (Yang and Rannala, 2006, Rannala and Yang, 2007, dos Reis and Yang, 2013, Zhu et al., 2015).

Our analyses highlight the fact that the different dating programs such as MrBayes, BEAST, and MCMCTree use different and somewhat arbitrary procedures to construct the prior on divergence times and the resulting time priors may be very different among the programs even if exactly the same fossil calibration information is specified. We suggest that the user should be aware of such differences and always inspect the time prior by running the program without using the sequence data. The differences in the time prior may and may not have a large impact on the posterior time estimates, depending on the number, nature and locations of the fossil calibrations on the phylogeny, the amount of sequence data, and the seriousness of the violation of the clock, among other things. Similarly it is not possible to make a general recommendation as to which procedure is more appropriate for all datasets (perhaps beyond the fact that the ‘multiplicative construction’ is a mathematical mistake and should be avoided). A procedure that produces time priors that better match the original calibration densities should make it easier for the user to summarize the fossil evidence, but we note that such a requirement may not be achievable because truncation can have a very large effect so that the effective priors are very different from the calibration densities whatever procedure is followed to convert the calibration densities to the effective time prior. In the future, we see probabilistic modeling and statistical analysis of fossil data (including both fossil presence/absence data and morphological measurements) as an important approach to summarizing the fossil evidence to generate distributions of divergence times for use as molecular clock calibrations (Tavaré et al., 2002, Wilkinson et al., 2011, Ronquist et al., 2012a, Bracken-Grissom et al., 2014, Heath et al., 2014). For the present, we suggest that the paleontologist should take a proactive role in constructing calibration densities, by making subjective judgments regarding the quality of the fossil and its placement on the phylogeny. We also encourage the use of the error probabilities in soft-bound calibrations as an approach to represent the uncertainties in the soft maximum bounds. It should be stressed that decisions will be made arbitrarily by the computer program if not subjectively by the paleontologist. Given that in many cases the resulting time prior can be quite counterintuitively different to the calibration densities, we cannot emphasize enough how important it is for the user to explicitly calculate the time prior by running the MCMC analysis without data.

In this paper, we have focused on divergence time estimation when fossil calibration information is available on certain nodes on the tree, a procedure called *node calibration*. Recently *tip-calibration* methods have been developed, which analyze fossil data jointly with molecular data, in the so-called fossilized birth-death process model (Heath et al., 2014, Zhang et al., 2016). Morphological characters for both extant and extinct (fossil) species can be incorporated into a joint analysis with the molecular data for extant species (Ronquist et al., 2012a, O'Reilly et al., 2015). The dates for the fossil species provide the calibration information that resolves the morphological distances into absolute times and rates, which are propagated to the other nodes on the phylogeny represented by the molecular data. While the approach shows great promise, it has its own set of challenges (dos Reis et al., 2016, Ronquist et al., 2016). First, morphological characters, driven by natural selection and adaptation to environment and occasionally undergoing convergent evolution, rarely evolving in a clock-like fashion (Kimura, 1983). Second, morphological characters may be strongly correlated. Thus current models (Lewis, 2011), which ignore the correlation, are overstating the information content in the data. Third, without constraints on the interior nodes, the Bayesian dating analysis tends to be very sensitive to the birth-death-sampling process used to specify the time prior. Changing the parameters in the branching process may change the shape of the tree (reflected in the relative of internal versus external branch lengths), leading to drastically different posterior time estimates (Drummond and Stadler, 2016, Ronquist et al., 2016, Zhang et al., 2016). We believe that both node calibrations and tip calibrations will have a major role to play in the foreseeable future (O'Reilly et al., 2015).

## Author contributions

M.d.R, and Z.Y. conceived the project and designed the analysis. J.B.-M. prepared the data sets and carried out the real data analysis. M.d.R carried out the theoretical 5-species analysis. All authors contributed to the interpretation of results and worked on the manuscript.
