# Supplementary material for: Comparison of different strategies for using fossil calibrations to generate the time prior in Bayesian molecular clock dating
Source: Mol Phylogenet Evol. 2017 Sep;114:386–400. doi: 10.1016/j.ympev.2017.07.005 (PMC5546266; doi:10.1016/j.ympev.2017.07.005)
Supplement: Supplementary data 1 [file mmc1.docx]

***Mol Phylogenet Evol***

Comparison of different strategies for using fossil calibrations to generate the time prior in Bayesian molecular clock dating

José Barba-Montoya^1^, Mario dos Reis^1, 2^ and Ziheng Yang^1^

1. *Department of Genetics, Evolution and Environment, University College London, Gower Street, London, WC1E 6BT, UK.*

2. *School of Biological and Chemical Sciences, Queen Mary University of London, Mile End Road, London, E1 4NS, UK*.

Correspondence: [z.yang@ucl.ac.uk](mailto:z.yang@ucl.ac.uk)

# Supplementary Material

## Supplementary Figures S1-S2 (pages 2-3)

## Supplementary Tables S1-S2 (pages 4-5)

## *Supplementary figures*


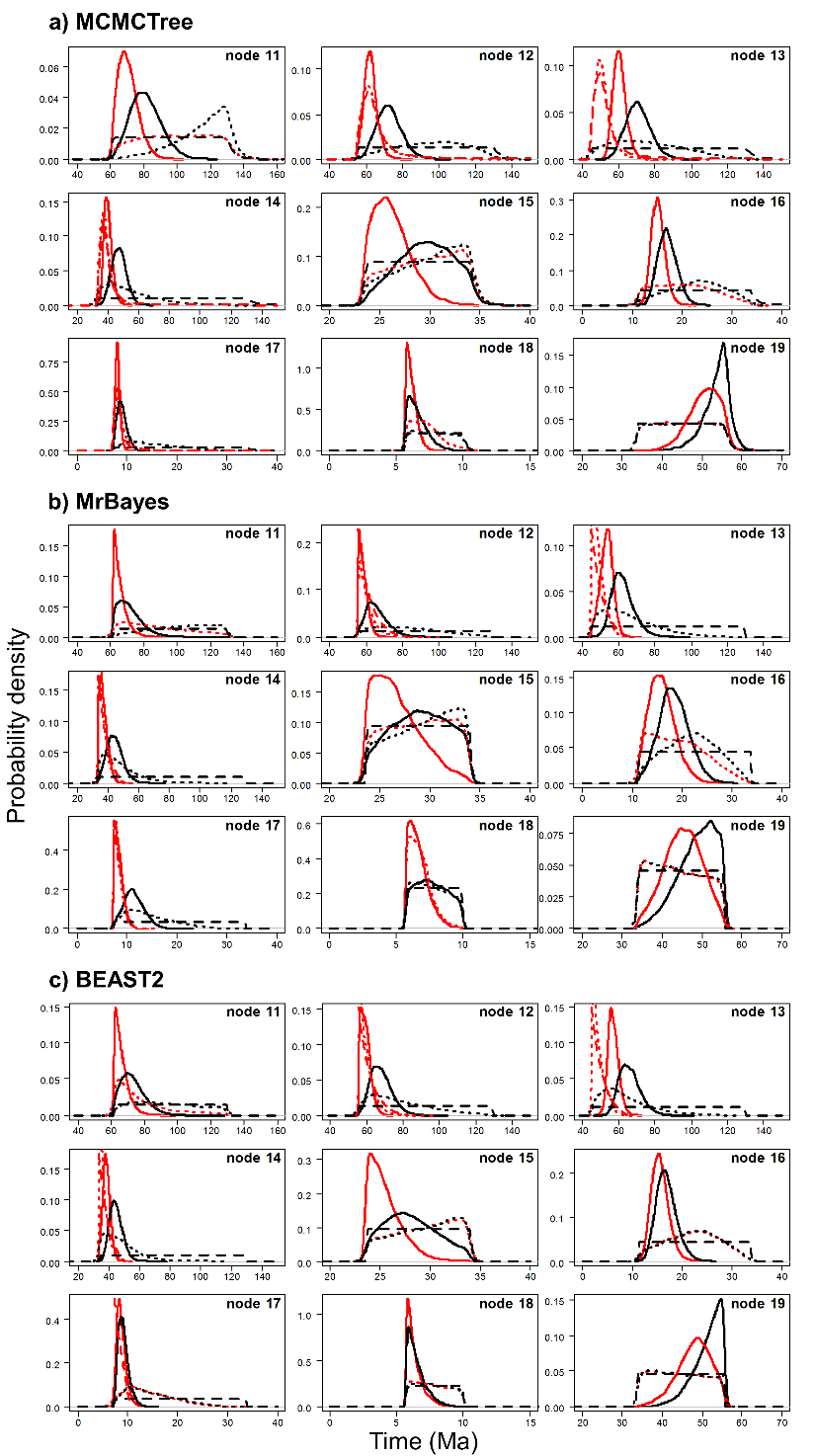


**Figure S1.** User-specified calibration densities (dashed lines), effective time priors (dotted lines), and the posterior (solid lines) for the primate dataset, under calibration strategies st1 (red) and st2 (black), implemented in MCMCTree, BEAST2 and MrBayes.


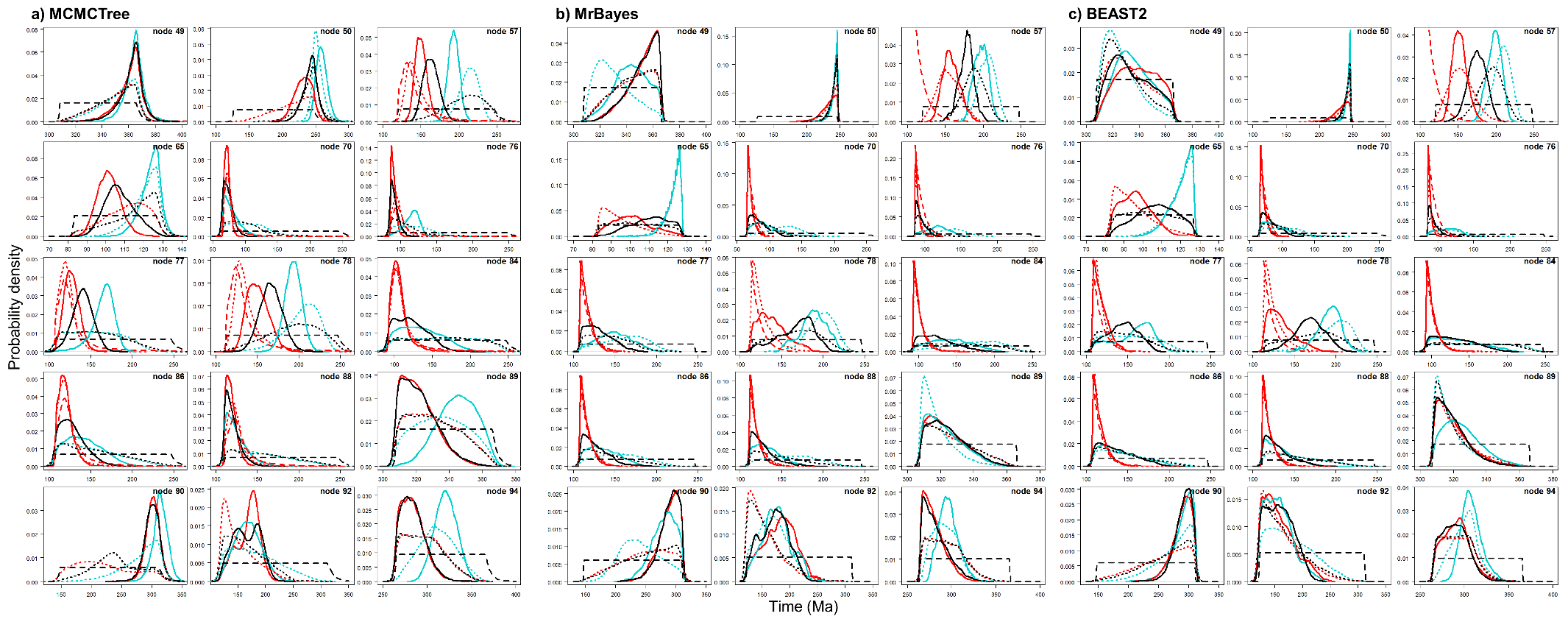


**Figure S2.** User-specified calibration densities (dashed lines), effective time priors (dotted lines), and the posterior (solid lines) for the seed plant dataset, under calibration strategies st1 (red), st2 (black), and st3 (blue), implemented in MCMCTree, BEAST2 and MrBayes. Only the 15 calibration nodes are used in the plots.

## *Supplementary tables*

**Table S1.** GenBank accession numbers of genes included in the primate dataset.

| Sampled taxa | Mitochondrial protein coding | | | | | | | | Mitochondrial rRNA | |
| --- | --- | --- | --- | --- | --- | --- | --- | --- | --- | --- |
|  | *Cyt B* | *CO1* | *CO2* | *CO3* | *ND2* | *ND3* | *ND4* | *ND4L* | *12S* | *16S* |
| *Callithrix jacchus* | AB572419 | AB572419 | AB572419 | AB572419 | AB572419 | AB572419 | AB572419 | AB572419 | AB572419 | AB572419 |
| *Gorilla gorilla* | NC_011120 | NC_011120 | NC_011120 | NC_011120 | NC_011120 | NC_011120 | NC_011120 | NC_011120 | NC_011120 | NC_011120 |
| *Homo sapiens* | NC_012920 | NC_012920 | NC_012920 | NC_012920 | NC_012920 | NC_012920 | NC_012920 | NC_012920 | JQ724861 | NC_012920 |
| *Macaca mulatta* | NC_005943 | NC_005943 | NC_005943 | NC_005943 | NC_005943 | NC_005943 | NC_005943 | NC_005943 | NC_005943 | NC_005943 |
| *Microcebus murinus* | GU327180 | EU179510 | GU326994 | AF224624 | — | AF224624 | AF224624 | AF224624 | AY582694 | AF072424 |
| *Otolemur garnettii* | AY441466 | AY671787 | — | — | — | — | — | — | DQ073511 | AF072430 |
| *Pan troglodytes* | NC_001643 | NC_001643 | NC_001643 | NC_001643 | NC_001643 | NC_001643 | NC_001643 | NC_001643 | NC_001643 | NC_001643 |
| *Pongo abelii* | NC_002083 | NC_002083 | NC_002083 | NC_002083 | NC_002083 | NC_002083 | NC_002083 | NC_002083 | NC_002083 | NC_002083 |
| *Tarsius syrichta* | AB371090 | AB371090 | AB371090 | AB371090 | AB371090 | AB371090 | AB371090 | AB371090 | AB371090 | AB371090 |
| *Tupaia belangeri* | NP_065227 | NP_065217 | NP_065218 | NP_065221 | NP_065216 | NP_065222.1 | NP_065224.1 | NP_065223 | NC_002521 | NC_002521 |

**Table S2.** GenBank accession numbers of genes included in the seed plant dataset.

| Sampled taxa | Plastid protein coding | | | | | Nuclear rRNA | |
| --- | --- | --- | --- | --- | --- | --- | --- |
|  | *atpB* | *matK* | *NdhF* | *rbcL* | *rps4* | *18s* | *26s* |
| *Acorus* | 156622714 | 69217282 | 62903246 | 37959585 | 69216101 | 1280175 | 2687430 |
| *Amborella* | — | 77743619 | 12005302 | 37194760 | 32401792 | 1777635 | 30527312 |
| *Arabidopsis* | NC_000932 | NC_000932 | NC_000932 | NC_000932 | — | 255689731 | — |
| *Austrobaileya* | — | 77743621 | 9623112 | 37194768 | AF313613 | 1022919 | 30527315 |
| *Berberidopsis* | HQ843255 | EU002171 | EU002201 | EU002274 | EU002295 | AF206866 | AF389242 |
| *Buxus* | 5001578 | 33333425 | 13491703 | 81230666 | — | 1369761 | 22595016 |
| *Cabomba* | — | 33333427 | 11022839 | 336459 | — | 470806 | 30527316 |
| *Calycanthus* | AJ235422 | AF543730 | AF123802 | L14291 | AY832286 | U38318 | 30527317 |
| *Ceratophyllum* | — | 33333433 | 6424767 | 6513623 | — | 2588926 | 30527319 |
| *Chloranthus* | — | — | 85740637 | 37194780 | 16565398 | 470845 | 19919686 |
| *Cornus* | AY725918 | 37935834 | 170178386 | 85678969 | 157689453 | 18108 | 85678959 |
| *Cycas* | 156597988 | 15866114 | 156597953 | 156598062 | 16565388 | 470889 | 66969255 |
| *Daucus* | 113200887 | 2281160 | 113200887 | 1374996 | 113200887 | — | 37778850 |
| *Dioscorea* | 17224736 | 10863023 | — | 17224611 | 6002044 | 194022474 | 18032178 |
| *Ginkgo* | 69214415 | 170320099 | 156598259 | 459408 | 125662705 | 471060 | 30527338 |
| *Gnetum* | — | 42529088 | — | 34733649 | 16565390 | 471074 | 2687428 |
| *Gunnera* | EU002162 | AM396506 | 157689283 | 11323502 | 157689481 | 1777743 | 22595023 |
| *Hedyosmum* | — | 89242559 | 85740639 | HQ336536 | — | 7595445 | 30527324 |
| *Ilex* | GQ997300 | EF590403 | 22796548 | EF590536 | GQ983972 | 7595458 | 19919644 |
| *Illicium* | — | 33333445 | 11022853 | 37194806 | 125662731 | 471848 | 66969247 |
| *Ipomoea* | EU118126 | EU118126 | EU118126 | EU118126 | EU118126 | 1049331 | 6707928 |
| *Leea* | AJ235520 | AF274621 | 157689307 | AJ235783 | 157689509 | AY674612 | 9799451 |
| *Liquidambar* | 157689159 | AF015651 | 157689309 | 86373225 | 157689513 | 471888 | 19919658 |
| *Liriodendron* | — | 7239757 | 6424765 | 13539638 | — | 471902 | 30527327 |
| *Magnolia* | — | 18025023 | 16416698 | 18024760 | 32401803 | 471957 | 22595029 |
| *Metasequoia* | 8439471 | 9279988 | 33416153 | 4049493 | 125662697 | 125662660 | 66969251 |
| *Morus* | 8439474 | 30421073 | 33950066 | 533039 | — | 532608 | 19919673 |
| *Musa* | 156598338 | GQ374866 | 156598303 | 342515 | 156598367 | 125661879 | 27462207 |
| *Nandina* | 904133 | 23495303 | 24934989 | 904135 | 32401801 | 904137 | 22595014 |
| *Nelumbo* | 194267386 | AM396514 | 193957791 | 229464449 | GQ997619 | 472018 | 22595032 |
| *Nymphaea* | — | 77743669 | 39598867 | 342748 | 32401794 | 472389 | 30527328 |
| *Oryza* | AB037543 | 7140883 | — | 344016 | — | 472190 | — |
| *Oxalis* | 257853489 | EU002186 | 157689327 | 257783291 | 157689531 | AF206978 | 19919671 |
| *Petalonyx* | 6689009 | 21464757 | 6706314 | 10945630 | JF268455 | 6688995 | 30230598 |
| *Pinus* | — | 148832433 | — | 86559793 | 16565394 | 472085 | 20467279 |
| *Platanus* | 194267388 | 166156329 | 193957793 | 86373193 | 32401796 | 472132 | 9799460 |
| *Podocarpus* | 33318668 | 259191248 | 33416155 | 33317783 | 32401837 | 20502 | 66969252 |
| *Populus* | AF209658 | EU749357 | AY757172 | EU676964 | — | DQ371807 | 19919559 |
| *Quercus* | 157689165 | 206604156 | 157689339 | 46091778 | 157689545 | 37729422 | 37993790 |
| *Spinacia* | AF528861 | NC_002202 | AY090621 | AJ400848 | AJ400848 | L24420 | HQ843464 |
| *Staphylea* | 157689167 | 157689209 | 84872975 | 57490179 | 157689559 | — | 19919574 |
| *Trimenia* | — | 89242601 | 37544941 | 7580491 | — | — | 30527333 |
| *Trithuria* | 21684894 | — | 33333926 | 118152381 | — | — | — |
| *Trochodendron* | 157689169 | 2149797 | 157689369 | 7240475 | 157689575 | — | 19919646 |
| *Vitis* | DQ424856 | AF274635 | DQ424856 | L01960 | DQ424856 | AF207053 | AF479207 |
| *Welwitschia* | — | 14579066 | — | 4049566 | 32401829 | 472502 | 110757094 |
| *Yucca* | 156622714 | AB088789 | 33333914 | 37722389 | 69216111 | 61741965 | — |
| *Zamia* | — | 15866124 | 33416161 | 32811545 | 32401756 | 475140 | 66969254 |
